# Attenuated huntingtin gene CAG nucleotide repeat size in individuals with Lynch syndrome

**DOI:** 10.1038/s41598-024-54277-5

**Published:** 2024-02-21

**Authors:** Karin Dalene Skarping, Larissa Arning, Åsa Petersén, Huu Phuc Nguyen, Samuel Gebre-Medhin

**Affiliations:** 1 Division of Clinical Genetics, Department of Laboratory Medicine, Lund University, Lund, Sweden; 2 Department of Clinical Genetics and Pathology, Office for Medical Service, 221 85 Lund, Sweden; 3 Translational Neuroendocrine Research Unit, Department of Experimental Medical Science, Lund University, Lund, Sweden; 4 Department of Human Genetics, Faculty of Medicine, Ruhr University Bochum, Universitätsstr. 150, 44801 Bochum, Germany

**Keywords:** Huntington disease, Lynch syndrome, Mismatch repair, *HTT* CAG repeat size, Huntington's disease, DNA mismatch repair

## Abstract

DNA mismatch repair (MMR) is thought to contribute to the onset and progression of Huntington disease (HD) by promoting somatic expansion of the pathogenic CAG nucleotide repeat in the huntingtin gene (*HTT*). Here we have studied constitutional *HTT* CAG repeat size in two cohorts of individuals with Lynch syndrome (LS) carrying heterozygous loss-of-function variants in the MMR genes *MLH1* (*n* = 12/60; Lund cohort/Bochum cohort, respectively), *MSH2* (*n* = 15/88), *MSH6* (*n* = 21/23), and controls (*n* = 19/559). The sum of CAG repeats for both *HTT* alleles in each individual was calculated due to unknown segregation with the LS allele. In the larger Bochum cohort, the sum of CAG repeats was lower in the *MLH1* subgroup compared to controls (*MLH1* 35.40 CAG repeats ± 3.6 vs. controls 36.89 CAG repeats ± 4.5; *p* = 0.014). All LS genetic subgroups in the Bochum cohort displayed lower frequencies of unstable *HTT* intermediate alleles and lower *HTT* somatic CAG repeat expansion index values compared to controls. Collectively, our results indicate that MMR gene haploinsufficiency could have a restraining impact on constitutional *HTT* CAG repeat size and support the notion that the MMR pathway is a driver of nucleotide repeat expansion diseases.

## Introduction

Huntington disease (HD) is one of at least nine Mendelian CAG/polyglutamine diseases, in which CAG nucleotide repeat expansions encode elongated stretches of glutamines in the respective disease-associated protein^1^. In HD, the underlying pathogenic CAG repeat is located in exon 1 in the huntingtin gene (*HTT*)^2^. By not fully understood mechanisms during meiosis, pathogenic *HTT* CAG repeat expansions (≥ 36 CAG repeats) can arise from the transition of harmless but unstable intermediate alleles (27–35 CAG repeats) to HD incomplete-penetrance alleles (36–39 CAG repeats) or HD full-penetrance alleles (≥ 40 CAG repeats) upon inheritance^2^. The CAG repeat expansion in HD triggers a protracted cascade of events, leading particularly to the degeneration of medium-spiny neurons in the striatum but also neuronal loss in other brain regions, causing movement, cognitive and psychiatric disorders with a wide spectrum of signs and symptoms^3,4^. HD full-penetrance allele repeat size inversely correlates with the age of disease onset (AO)^5^ but does not fully explain AO variability. In addition to a variable CAA interruption of the *HTT* CAG repeat that modulates AO^6–8^, genome-wide association studies (GWAS) have also identified several DNA maintenance genes as modifiers of AO including genes encoding components of the DNA mismatch repair (MMR) pathway^9–13^ (for review, see^14^). The MMR pathway is involved in the correction of misaligned DNA strands, the event which frequently occurs in genomic regions with mono- di-, or trinucleotide repeats (also termed short tandem repeats; STRs or microsatellites) in both replicating and non-replicating cells^15^. There is now a large body of experimental evidence showing that MMR contributes to the AO and disease progression in HD by promoting somatic expansion of the pathogenic CAG repeat, highlighting the MMR pathway as a potential target for therapeutic interventions^14^. Individuals heterozygous for germline loss-of-function (LoF) pathogenic variants in either of the MMR genes *MLH1*, *MSH2*, *MSH6* or *PMS2* have Lynch syndrome (LS), an autosomal dominant predisposition mainly to colorectal cancer and endometrial cancer (for a recent review of LS, see^16^). The biochemical hallmark of LS cancer is deficient MMR (dMMR) due to somatic inactivation of the wild type allele of the affected MMR gene, and as a consequence LS cancer cells show immunohistochemical loss of MMR protein expression and accumulate nucleotide repeat aberrations known as microsatellite instability (MSI). In gametes of individuals with LS, the state of haploidy at meiosis implies that oocytes and sperm cells carrying MMR LoF alleles are subject to dMMR. In the present study we therefore hypothesized that MMR gene haploinsufficiency could affect constitutional *HTT* CAG repeat size. To test this hypothesis, we determined *HTT* CAG repeat size in individuals with LS.

## Results

### Characterization and analysis of the Lund cohort

**Figure 1:**
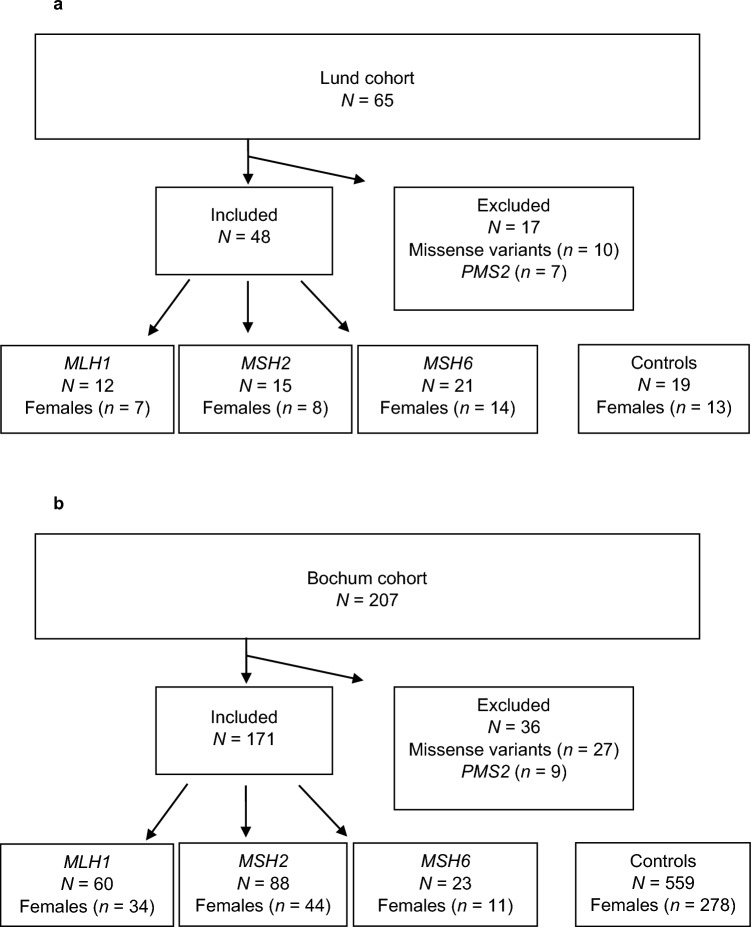
Flow-chart and description of the Lund cohort (**a**) and the Bochum cohort (**b**) with numbers of included and excluded individuals, gender distribution, and Lynch syndrome genetic subcategories with loss-of-function variants in *MLH1*, *MSH2* and *MSH6*, respectively, and controls.

**Figure 2:**
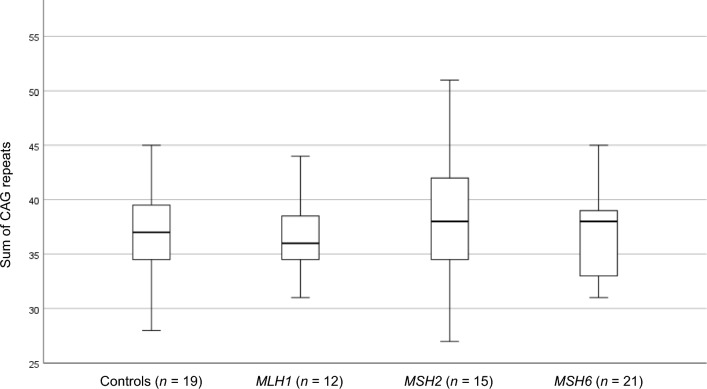
Boxplot of the sum of CAG repeats in the Lund cohort from individuals with Lynch syndrome caused by loss-of-function variants in *MLH1*, *MSH2* and *MSH6*, and controls. Outlier (*MLH1 n* = 1, 55 CAG repeats) is not shown.

Initially, a cohort of individuals with LS (*n* = 65) was investigated (Lund cohort; Fig. 1 a). A total of 48 individuals with LoF variants in either of *MLH1* (*n* = 12), *MSH2* (*n* = 15) and *MSH6* (*n* = 21) were subjected to constitutional *HTT* CAG repeat size estimation and compared with controls (*n* = 19) (Fig. 1a). The sum of CAG repeats did not differ significantly between individuals with LoF variants in *MLH1* (37.75 CAG ± 6.3; mean ± SD), *MSH2* (37.93 ± 5.8), *MSH6* (36.95 ± 4.0) and controls (36.84 ± 4.4) (Fig. 2; Table 1). Three individuals had one *HTT* allele with CAG repeats in the intermediate allele interval (*MLH1*, *n* = 1, *MSH2*, *n* = 1, controls, *n* = 1; Table 1). The remaining alleles were in the normal allele interval (≤ 26 CAG repeats).

**Table 1:** Summary of *HTT* CAG repeat size characteristics in the study cohorts.

Group of individuals	Sum of CAG repeats (mean ± SD)	Fraction (%) of individuals with 27–35 CAG repeats	Mean somatic EI value
**Lund cohort**			
Controls	36.84 ± 4.4	1/19 (5.3)	NA
*MLH1*	37.75 ± 6.3	1/12 (8.3)	NA
*MSH2*	37.93 ± 5.8	1/15 (6.7)	NA
*MSH6*	36.95 ± 4.0	0/21	NA
**Bochum cohort**			
Controls	36.89 ± 4.5	29/559 (5.2)	0.131
Without IA	36.34 ± 4.0	0/530	0.116
*MLH1*	35.40 ± 3.6*	1/60 (1.7)	0.099
Without IA	35.19 ± 3.2**	0/59	0.096
*MSH2*	36.41 ± 5.2	3/88 (3.4)	0.122
*MSH6*	36.17 ± 4.2	1/23 (4.4)	0.100

The mean sum of *HTT* CAG repeats, the fraction of individuals with *HTT* intermediate alleles (27–35 CAG repeats), and the mean somatic expansion index (EI) value in the *MLH1*, *MSH2* and *MSH6* Lynch syndrome subgroups and controls in the Lund cohort and Bochum cohort, respectively, are shown. SD, standard deviation. NA, not analyzed. IA, individuals with *HTT* intermediate alleles. *P = 0.014 for *MLH1* subgroup/controls. **P = 0.031 for *MLH1* subgroup without IA/controls without IA.

### Characterization and analysis of the Bochum cohort

**Figure 3:**
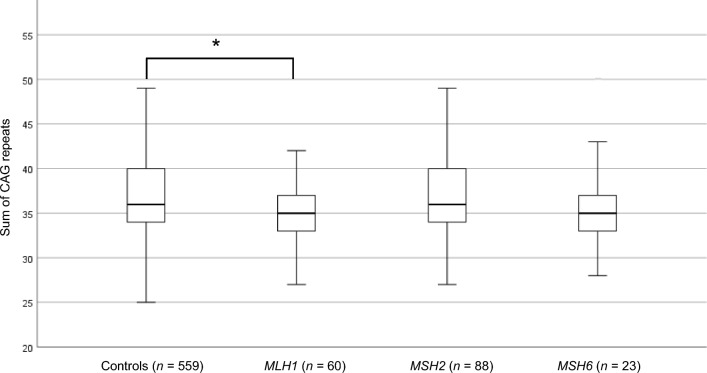
Boxplot of the sum of CAG repeats in the Bochum cohort from individuals with Lynch syndrome caused by loss-of-function variants in *MLH1*, *MSH2* and *MSH6*, and controls. Outlier (*MSH6 n* = 1, 50 CAG repeats) is not shown. *P = 0.014.

Subsequently, a larger cohort of individuals with LS (*n* = 207) was investigated (Bochum cohort; Fig. 1b). A total of 171 individuals (mean age 47.1 ± 12.9 years) with LoF variants in either of *MLH1* (*n* = 60; mean age 47.6 ± 13.1 years), *MSH2* (*n* = 88; mean age 46.6 ± 13.1 years) and *MSH6* (*n* = 23; mean age 48 ± 11.5 years) were subjected to constitutional *HTT* CAG repeat size estimation and compared with controls (*n* = 559; mean age 27.3 ± 9.4 years) (Fig. 1b). The sum of CAG repeats in individuals with LoF variants in *MLH1* was significantly smaller than in controls (35.40 CAG ± 3.6 vs. 36.89 CAG ± 4.5 in controls, Student’s t-test with Bonferroni correction, *p* = 0.014, CI − 2.766 to − 0.310) (Fig. 3; Table 1) and remained significantly smaller also after removal of individuals with *HTT* intermediate alleles in an additional analysis (35.19 CAG ± 3.2 vs. 36.34 CAG ± 4.0 in controls; Student’s t-test, *p* = 0.031, CI − 2.202 to − 0.104) (Table 1). The sum of CAG repeats in individuals with LoF variants in *MSH2* (36.41 CAG ± 5.2) and *MSH6* (36.17 CAG ± 4.2) did not differ significantly from controls (Fig. 3; Table 1). Thirty-four individuals had one *HTT* allele with CAG repeats in the intermediate allele interval (*MLH1 n* = 1, *MSH2 n* = 3, *MSH6* *n* = 1, controls *n* = 29; Table 1). The remaining alleles were in the normal allele interval. The fraction of individuals with an intermediate allele among individuals with LS did not differ significantly from that in controls, but the fraction was consistently lower in all LS genetic subgroups (Table 1). The mean somatic *HTT* CAG expansion index (EI) value, which typically is increased in tissues from individuals with HD^17^, did not differ significantly between individuals with LoF variants in *MLH1* (EI = 0.099), *MSH2* (EI = 0.122), *MSH6* (EI = 0.100) and controls (EI = 0.131) (Table 1). However, notably all LS genetic subgroups showed a lower mean EI value compared to controls (Table 1).

## Discussion

Investigation of *HTT* CAG repeat size in lymphocyte DNA from 217 individuals from two different LS cohorts, showed a small but statistically significant CAG repeat size reduction in a subgroup of 60 *MLH1* LoF heterozygotes from the larger cohort from Bochum. The frequencies of *HTT* intermediate alleles and somatic EI values were consistently lower in all LS genetic groups compared to controls in the Bochum cohort, but the observed differences were individually not statistically significant. The CAG repeat size in the Bochum *MLH1* LS subgroup remained significantly smaller compared to controls also after removal of individuals with *HTT* intermediate alleles. Nucleotide repeat instability and an increased mutational burden is a known phenomenon in dMMR cancers in LS patients following somatic “second hit” of the remaining wild-type MMR allele^16^, and in all tissues in individuals with constitutional biallelic MMR deficiency^18^. However, to the best of our knowledge, MMR gene haploinsufficiency in humans has to date not been reported to affect constitutional nucleotide repeat size. A recent whole genome sequencing (WGS) study of non-neoplastic tissue samples from individuals with LS failed to detect any changes in the repertoire of mutational processes or mutation rates^19^. Yet, subtle nucleotide repeat variations could have escaped detection using WGS technology due to limited methodological accuracy in regions with STRs compared to PCR fragment-based analyses^20^. Like previous population-based observations^21^, we found a large CAG repeat size variation between *HTT* alleles, both intra- and inter-individually which, together with the lack of parental *HTT* repeat size data prevent us from a more detailed data interpretation. Clearly, the deciphering of which *HTT* allele has cosegregated with the LS allele in each individual, e.g., by LS family trio analyses, would have enhanced our data interpretation considerably, allowing us to identify individuals, or certain repeats size intervals including variable CAA interruptions that may account for the observed repeat variation in the Bochum LS cohort. Although *HTT* intermediate alleles appear under-represented in the Bochum LS cohort, especially in individuals with *MLH1*-associated LS (1.7%), compared to the controls used (5.2%) and to reported population-based frequencies (6.8%;^21^), interpretation of data should be made with caution due to the limited size of the cohort subgroups and the absence of LS family trio data. Possibly, the observed frequency of *HTT* intermediate alleles in LS in our study could reflect intergenerational CAG repeat contractions of such alleles into the normal repeat-size interval. However, *HTT* intermediate alleles alone are not responsible for the observed CAG repeat-size reduction in the Bochum *MLH1* LS subgroup as the removal of this category of alleles from our calculations had little impact. Somatic EI values did not differ significantly between the LS subgroups and controls, but notably the values were consistently lower in all LS genetic subcategories. As EI values normally are positively age-dependent^22^ and since the mean age in the Bochum LS group was higher than in the control group, the EI value gap between the two groups could potentially be an underestimate. Clearly, the use of age-matched controls would have sharpened interpretation of EI values. Experimentally, in mouse models of HD, there is long-standing evidence that reduced expression of the MMR proteins Msh2, Msh3, Mlh1 or Mlh3 counteracts somatic CAG repeat expansion^23,24^ and de-escalates the HD experimental pathogenic process^25^. More recently, reduced expression of the endo- and exonuclease Fan1 was shown to promote somatic CAG repeat expansion in an Mlh1-dependent manner, i.e., suppression of Mlh1 blocked Fan1-induced repeat expansion^26,27^. There is now mounting evidence that the MMR pathway contributes to the expansion of unstable pathogenic nucleotide repeats in HD and other human hereditary neurodegenerative diseases^14^. Given the present results and current knowledge in this field of research, it could be speculated that individuals with LS could be less prone to *HTT* CAG repeat expansion. In summary, this study indicates that MMR gene haploinsufficiency, in particular for *MLH1*, could be associated with a propensity for reduced constitutional *HTT* CAG repeat size. Further investigations, e.g., with larger LS case samples and LS family trio WGS analyses are required to confirm our results. Additional studies should also be encouraged to explore the possible impact of MMR gene haploinsufficiency on other nucleotide repeat regions in the human genome.

## Methods

### Cohort information

Lymphocyte DNA was retrieved from two different cohorts of index individuals diagnosed with LS from Sweden and Germany (Lund cohort and Bochum cohort, respectively) carrying germline class 4 (likely pathogenic) or class 5 (pathogenic) variants in *MLH1*, *MSH2*, *MSH6* and *PMS2* according to variant classification criteria by The American College of Medical Genetics and Genomics (ACMG)^28^ or The International Society of Gastrointestinal Hereditary Tumours variant database^29^, and from controls (Fig. 1). A subgroup of the Lund cohort was previously presented in a pre-publication (Dalene Skarping et al. 2022, MedRxiv, 10.1101/2022.05.28.22275723). Controls in the present study were individuals diagnosed with immunohistochemically MMR proficient colorectal cancers during 1999–2011 from whom tumor tissue DNA had also been archived (Lund cohort) or self-reported healthy university students (Bochum cohort). Controls from Bochum were excluded if they or any of their close relatives suffered from neurological and/or mental illnesses, as assessed by a self-report questionnaire. Individuals with LS-associated missense variants predicted to cause single amino acid substitutions were excluded to avoid variants with partial LoF, and variants with unclear pathogenic mechanism. Other types of LS-associated variants, i.e., nonsense variants, variants altering the reading frame or splicing, deletions or duplications of exon(s) were considered complete LoF alleles. Individuals with variants in the MMR gene *PMS2* were excluded due to the limited number of such individuals in both cohorts (Fig. 1).

### *HTT* CAG repeat size estimation and somatic expansion ratio calculation

*HTT* germline CAG repeat size estimation was performed using standard protocols for PCR amplification and capillary electrophoresis fragment analysis with a validated accuracy of ± 1 CAG repeat for alleles with < 45 repetitions and ± 3 CAG repetitions for alleles with 45 or more repeats using PCR primers (Lund cohort) HD1: 5′ ATGAAGGCCTTCGAGTCCCTCAAGTCCTTC 3′ and HD3: 5′ Hex-GGCGGTGGCGGCTGTTGCTGCTGCTGC 3′ as described^30^, or (Bochum cohort) Hu4: (F) 6-FAM-5′-ATGGCGACCCTGGAAAAGCTGATGAA) and Hu5: (R) (5′-GGCGGTGGCGGCTGTTGCTGCTGCTGCTGC) as described^31,32^. A canonical glutamine-encoding repeat sequence in *HTT* was assumed. PCR products were resolved using the ABI 3500XL Genetic Analyzer (Applied Biosystems) using GeneMapper v6 software and GeneScan 500-ROX as internal size standard (Lund cohort), or ABI 3500XL Genetic Analyzer (Applied Biosystems), GeneMapper v4.1 software and GeneScan 500-ROX as internal size standard (Bochum cohort). Somatic CAG repeat EI values were derived from indices from GeneMapper peak height data and calculated as described^17^, considering only expansion peaks to the right of the highest (modal allele) peak, using 250 consecutively selected individuals from the Bochum control group as controls.

### Statistical analyses

CAG repeat size was converted to integers according to clinical genetic laboratory diagnostic routines^30^. The methodological estimation error ± 1 repeat was excluded from statistical calculations. Since the methods used in this study do not unmask which *HTT* allele has co-segregated with the LS-associated variant, the sum of *HTT* CAG repeats in each individual was calculated and used in all analyses except for somatic EI calculations. Mean values for sum of CAG repeats and standard deviation (SD) with 95% confidence interval (CI) were calculated for each MMR gene. Student’s t-test was used. *P-*values < 0.05 were considered significant. Bonferroni correction was applied to adjust for multiple comparisons, i.e., *MLH1*, *MSH2* and *MSH6* vs. controls, respectively, following which *P-*values < 0.017 were considered significant. Calculations were performed using SPSS Statistics for Windows (SPSS Inc., Chicago, Ill., USA).

### Ethics approval

This study was approved by The Regional Ethical Review Board in Lund, Sweden (application no. 2013/468 and application no. 2015/211), approved, or waived following anonymization procedures by the Swedish Ethical Review Agency (application no. 2019-02312 and application no. 2021-06254-02, respectively), and approved by the Ethics Review Board of the Ruhr University in Bochum, Germany, (application no. 18-6563-BR). Informed written consent was required and obtained from all individuals (Bochum cohort) or waived (Lund cohort) following anonymization of DNA samples prior to *HTT* CAG repeat size analysis (application no. 2021-06254-02). No individual-level data are published in this study. All methods were performed in accordance with the relevant local guidelines and regulations.

## Data Availability

This manuscript contains all relevant data to the study. The raw case data sets generated and/or analyzed during the current study are not openly available due to ethical considerations related to patient privacy. Access to raw data and detailed protocols may be considered on request to the corresponding authors SGM or HPN.
